# A prescription for sustaining community engagement in malaria elimination on Aneityum Island, Vanuatu: an application of Health Empowerment Theory

**DOI:** 10.1186/s12936-015-0779-z

**Published:** 2015-07-31

**Authors:** Noriko Watanabe, Akira Kaneko, Sam Yamar, George Taleo, Takeo Tanihata, J Koji Lum, Peter S Larson, Nelma BC Shearer

**Affiliations:** Department of Parasitology, Osaka City University Graduate School of Medicine, Osaka, Japan; Department of Microbiology, Tumor and Cell Biology, Karolinska Institutet, Stockholm, Sweden; Ministry of Health, Port Vila, Vanuatu; Department of Infectious Disease Control, Healthcare Centre of Kobe, Kobe, Japan; Department of Anthropology and Biological Sciences, Binghamton University, Binghamton, NY USA; Nagasaki University Institute of Tropical Medicine, Nagasaki, Japan; University of Michigan School of Natural Resources and Environment, Ann Arbor, MI USA; Hartford Centre of Gerontological Nursing Excellence, College of Nursing and Health Innovation, Arizona State University, Phoenix, AZ USA

**Keywords:** Malaria elimination, Community engagement, Empowerment, Coalitions, Social networks, Social capital, Aneityum Island, Sustainability, Capacity, Local resources

## Abstract

**Background:**

Community engagement has contributed to disease control and elimination in many countries. Community engagement in malaria elimination (ME) on Aneityum Island has been sustained since its introduction in the early 1990s. Capacity developed within this population has led to a health empowered community response. Health Empowerment Theory (HET) can account for the innovative community actions and capacity development efforts taken to realize and sustain meaningful changes in well-being. This study used the HET framework to investigate participant perceptions of ME efforts on the island focusing on two HET elements, personal and social-contextual resources. The purpose of this study was to explore the role of empowerment as a critical element of community engagement.

**Methods:**

Six focus group discussions, ten key informant interviews and 17 in-depth interviews were conducted in July 2012 on Aneityum. Both deductive and inductive approaches to qualitative content analysis were used to identify themes, which were condensed, coded and classified based on the HET elements above.

**Results:**

Awareness and use of personal and social-contextual resources played an important role in ME efforts. Most participants shared their knowledge to prevent malaria reintroduction. Many participants reported their skills needed for behavioral maintenance, problem-solving or leadership. Participants who perceived a threat took preventive actions even in the dry season. Community leaders focused on second generation capacity development. A local health coalition provided ME services. Members of networks were sources of information and assistance. Face-to-face was the preferred method of communication. Barriers to engagement (e.g., financial difficulties, health literacy issues and underdeveloped infrastructure) were minimized through active collaboration and mutual assistance.

**Conclusions:**

In the community engagement continuum, health empowerment develops incrementally overtime as people gain their knowledge and skills, form coalitions and develop collaborative networks (social capital) to make decisions and take action for change. Community engagement, which facilitates local personal and social-contextual resource development, has potential for ME and multilevel empowerment through community-based capacity development processes. These self-empowered communities have written and will continue to write a ‘prescription’ for sustaining high levels of engagement.

**Electronic supplementary material:**

The online version of this article (doi:10.1186/s12936-015-0779-z) contains supplementary material, which is available to authorized users.

## Background

Community engagement has contributed to disease control and elimination in many countries [1–10]. Community engagement used in health interventions is recognized as the process of working collaboratively with and through groups of people affiliated by geographic proximity, special interests or similar situations to address issues affecting their well-being [9, 10]. Community engagement can only be sustained by developing the community’s capacity and resources and mobilizing community assets and strengths to make decisions and take action [9, 10]. The community engagement continuum consists of five levels toward greater community ownership and leadership [9]. ‘Outreach’ to the community is the lowest form in the continuum for one-way communication [9]. At the level of ‘consultation,’ the community is invited to present their perspectives or provide their feedback on matters of interest to health professionals [9]. At the level of ‘cooperation,’ community members cooperatively address community issues and eventually form a partnership [9]. At the ‘collaboration’ level, the communication flow is bidirectional, and partnership actions are taken on each aspect of the program [9]. ‘Shared leadership’ is the highest level of community engagement where the community shapes communication, makes decisions and takes initiatives [9].

Islands provide potential for intervention studies [11]. Aneityum, the southernmost island in Vanuatu offers a blueprint for sustained community engagement in malaria elimination (ME) over the last two decades [5, 11–14]. The elimination program on Aneityum that used mass drug administration, insecticide-treated nets and larvivorous fish (*Gambusia* spp.) was initiated in 1991 on Aneityum Island where transmission of *Plasmodium falciparum* and *Plasmodium vivax* was considered hypo- to meso-endemic at that time [5, 11–14]. At the beginning of the program, community engagement in ME was observable at the consultative stage to ensure compliance [5, 9]. Through intensive health education and promotion, the target population were kept informed of the actions taken to eliminate malaria (e.g., drug intake, use of nets and introduction of larvivorous fish) [5]. In the meantime, planners incorporated community input (e.g., local information, knowledge, organizational practices, coordinating structures, service-delivery structures, communication flows, communication channels and networks) into their plans [5, 11]. This early engagement process facilitated interrelated individual and structural forms of capacity development and resource mobilization for ME on the island. In 1992, the communities started to generate ME resources (e.g., village health volunteers trained specifically in malaria microscopy) [11]. In 2002, a ‘collaborative’ intervention effort between the provincial government, a foreign malariologist and communities quickly brought the recent malaria outbreak under control [11]. Up to now, six villagers largely motivated by intrinsic factors (e.g., feeling of making a contribution to the communities and skills gained) have guarded against imported malaria [11], suggesting that the communities have managed their resources to gain control over their lives and environment in the process of engagement. Through these actions, it is recognized that community engagement has moved from the initial level of ‘consultative’ engagement to ‘cooperative’ to ‘collaborative’ engagement on Aneityum [5, 9–11]. As individuals, organizations and communities move closer to the end of the engagement continuum as change agents rather than targets for change, the greater empowerment grows across individual, organizational and community levels [9]. Aneityum experience implies that empowerment has emerged from community efforts undertaken since the 1990s.

This study highlights the overlooked element behind the scenes of the Aneityum experience. The main point is that community engagement is sustained on Aneityum because it has been built on the process of enhancing the capacity and strength of individuals, organizations and communities to mobilize and manage resources locally. Therefore, community empowerment should not be studied in isolation from the ME processes or strategies. The purpose of this study was to explore the role of empowerment as a critical element of community engagement.

The authors of the Alma Ata Declaration presume that community members need to be empowered to fulfil their role as active participants [4]. Disease control programs are mostly built on individual and structural forms of capacity development [4]. Health Empowerment Theory (HET) can account for innovative community actions and individual and structural capacity development efforts taken to maintain health and wellbeing [15]. HET suggests that health empowerment emerges from a synthesis of personal and social-contextual resources including social networks and services [15]. This study used the HET framework to investigate participant perceptions of ME efforts on the island, focusing on two HET elements, personal and social-contextual resources.

## Methods

### Study settings

Aneityum is the only malarious island outside the Buxton line, which defines the south-eastern limit of anopheline breeding [11, 16]. Aneityum has an area of 159.2 sq. km. The total population of Aneityum is 915, of which 47% is female, and 53% is male, with 53% of the population age 0–19 years (2009 National census). Main villages include Analgaut, Port Patrick and Unmet [5, 11]. This study was carried out in July 2012 in the villages around Analgaut. The study island and villages were purposefully selected to capture the views of those living on the island where ME efforts were sustained over the past two decades. Residents were notified prior to the arrival of the study team by community leaders and local facilitators who had long cooperated with the communities to eliminate malaria.

### Data collection procedures and study participants

Qualitative methods were used, including Focus Group Discussions (FGDs), Key Informant Interviews (KIIs) and In-Depth Interviews (IDIs). Two males and two females were chosen to work as local facilitators. The first author, who had experience conducting FGDs, KIIs and IDIs to investigate community perceptions and practices relating to malaria prevention on Guadalcanal Island in Solomon Islands, was the main interviewer and moderator for all data collection methods with local facilitators/translators. After the first author explained the purpose of this study that required sufficient variation among participants to contrast opinions, participants for focus groups (i.e., all-female, all-male and mixed groups aged 16–31 years) were recruited by local facilitators through their local networks. FGDs with older participants (aged 32 years and above) could not be conducted due to a sporting event on the island and time constraints faced by researchers. To manage potentially challenging group dynamics (e.g., consensus view, distraction or unconscious manipulation), the discussants were divided into small groups (five discussants per each group). Key informants included *kastomary* chiefs (customary chiefs), teachers, religious leaders, health committee members, health care workers and shopkeepers. These informants were purposefully chosen because of their positions and the depth of their experience in the communities. IDI interviewees were recruited using purposive sampling to ensure diversity. Interviewees were chosen based on age, gender and place of residence (e.g., village).

Interview questions (see Additional file 1) were designed to capture two elements of HET (i.e., personal and social-contextual resources) [15]. Interview questions on personal resources were related to malaria risk perception, motivation and preventive health behavior (see Additional file 1). Interview questions on social-contextual resources were related to services and support networks. In order to explore support networks, participants selected for in-depth interviews were also asked to provide detailed information about people outside their home whom they would turn to for help if it was needed. Additionally this in-depth interview group was asked about their place of residence, in order that walking times could inform study analysis. Basic demographic data of participants including age, education and religious affiliation was recorded.

A researcher asked questions in English, and local facilitators simultaneously translated them from English to Bislama, and vice versa. Where this was not the case, research activities were carried out in English. On Aneityum, Bislama, English, French and local languages are spoken. Among the population aged five years and older, 64% of females are literate in Bislama and English, while 65 and 61% of males are literate in Bislama and English, respectively (2009 national census). 28% of females and 33% of males are literate in French (2009 national census).

Among the 57 study participants, 30 took part in the female and male FGDs (6 groups), ten in KIIs and 17 in IDIs (Table 1). Males comprised 42.1% of all participants (Table 2), because a few males refused to participate in the qualitative study due to their activities such as fishing or farming. Interviewees included six villagers living in rural or remote areas. Two female interviewees were single mothers. Two other female interviewees noted that they recently moved from other islands for marriage. Most participants represented Christian denominations, such as Presbyterian, Seventh-day Adventist and Catholic (Table 2). All participants had attended primary school, while 57.9% had attended secondary school (Table 2). Some elderly participants were functionally illiterate.

**Table 1:** Sample size

	Venue	Interview length (min)	Sample size	Age range
Focus Group Discussions	Community house	45	6 groups including 3 female youth groups 2 male youth groups 1 mixed youth group Total: 30 discussants	16–31
Key Informant Interviews	Dispensary	30–45	10 informants including 5 females 5 males	21–65
In-Depth Interviews	Community ground	20–120	17 interviewees including 9 females 8 males	16–67

**Table 2:** Demographic characteristics of participants

N = 57	N	Percentage of participants (%)
Gender		
Female	33	57.9
Male	24	42.1
Age group		
<20	17	29.8
20–40	27	47.4
41–60	9	15.8
>60	4	7.0
Education		
Primary	57	100.0
Secondary	33	57.9
Post-secondary	4	7.0
Religious affiliation		
Presbyterian	34	59.6
Seventh-day Adventist	11	19.3
Catholic	7	12.3
Unspecified	5	8.8

### Data analysis

Each interview was simultaneously translated and transcribed. The manuscript was shared and discussed among local facilitators to confirm the transcription and obtain their feedback. Because HET served as the main framework for this study, its two key elements (i.e., personal and social-contextual resources) served as the pre-existing categories. Therefore, deductive content analysis was applied [17–19]. Then, interview transcript was read several times to inductively identify common themes emerging from the data [17–19].

### Ethical considerations

This research was approved by the Vanuatu Ministry of Health and the Institutional Review Board of State University of New York at Binghamton (#1578-10).

Written or verbal consent to collect and publish data was obtained from all participants prior to commencing the research activity. Participants included two adolescents (aged 16 and 17 years) accompanied by caretakers. All participants were assured that their responses would remain confidential.

## Results

Themes that were inductively presented within the pre-existing categories of HET (i.e., personal and social-contextual resources) demonstrated individual and structural forms of capacity to promote the use of personal and social-contextual resources. These themes are presented below.

### Participant perceptions related to personal resources

Knowledge and skills led to increased ME efforts.

#### Theme 1: Knowledge

There were no differences between men and women in their reporting of knowledge of malaria and prevention measures. Older participants aged 32 and over were more likely than young participants who did not experience endemic malaria to have a deeper experience-based knowledge. Some elderly participants noted that they shared their experience and knowledge with young people.

Malaria was commonly considered dangerous and even life-threatening. Knowledge gained through a previous history of malaria encouraged some participants (including youth participants who had malaria on the other islands such as Efate and Tanna) to engage in ME activities. Some typical comments were:

“I am very afraid of malaria because I was infected with *P. vivax *in 2002 on Tanna Island. I had a high fever and sever pain. So, I go to the dispensary right away when I have a fever.” (Female KII, Analgaut)

“I was diagnosed with *P. vivax* at Vila when I was 14 years old. I am afraid of malaria. Malaria causes headache, fever and aches. Malarial fever is different from other fevers. I try to prevent malaria. I have a spare net for future use.” (Female IDI, Analgaut)

Many participants acknowledged potential risk factors on the island or other islands. They feared that imported malaria, which might be brought into by humans (e.g., islanders traveling outside of Aneityum Island, foreigners and strangers) and mosquitoes, could cause the outbreak on the island again. To minimize risk, participants reportedly engaged in ME efforts (e.g., the use of nets, cleanliness and early presentation for all fever episodes). A few elderly female participants even stated that foreign tourists or strangers should stay at Mystery Island (an uninhabited sandy islet with beaches and one airport), away from main land to prevent diseases. An elderly male interviewee reported the impact of tourism such as the introduction of new diseases, spread of Western lifestyles and destruction of local cultures. Some older participants who noted that foreigners or tourists would bring epidemic diseases to Aneityum reported consistent use of nets throughout the year. Several informants stated that they were able to control breeding sites or prevent mosquito bites. But they also reported that they were not able to control the movement of people.

“We can avoid mosquito bites, but we cannot control people. So, everyone on this island should be tested more frequently to maintain a healthy environment, because many strangers walk around the village.” (Male KII, Analgaut)

A few elder key informants noted that knowledge of health benefits gained through their experience encouraged participants to sustain the miracles that they saw in the 1990s. A few elderly interviewees and some young discussants did not express enthusiasm for ME activities. They insisted that nobody died of malaria for a long time on the island. Tangible benefits from tourism did not inspire participants to engage in ME activities. Some typical comments were:

“We do not think a malaria-free environment will increase the number of tourists. We do not see the relationship between a malaria-free island and tourism. Why do you ask?” (Female FGD, Analgaut)

“We prevent malaria for ourselves. We do not prevent malaria for foreigners.” (Male KII, Analgaut)

#### Theme 2: Skills

Skills including those needed for behavioral maintenance, problem-solving and leadership were widely reported by participants.

*Skills necessary to maintain the behavior change* Most participants, regardless of age or gender, reported being motivated to prevent malaria for their health and development. Several key informants noted that people on the island maintained a pattern of behavior. A few female informants stated that the access to and use of intervention services helped sustain changes since the 1990s. Many participants, regardless of age or gender, reported sleeping under a net even during the dry season when mosquito breeding sites are not abundant. Village and household cleanliness were frequently reported as essential for ME. All participants reported using Western medicine to treat malaria. All participants noted that they recognized the beneficial effect of Western medicine through the interventions and they preferred to use it rather than *kastom* medicine (traditional or herbal medicine). Some participants stated that they used *kastom* medicine only for various symptoms. Rapid diagnostic tests (RDTs) were not utilized by participants. All participants noted that they preferred malaria diagnosis using microscopy for a high fever. A few participants reported delays due to weather and community infrastructure.

“Three to five times a week, people turned up late for a test because of bad weather and location.” (Male KII, Analgaut)

“When it rains hard, it is difficult to go to the main village. I will check my blood later.” (Male IDI, Port Patrick)

*Problem*-*solving skills* A group of young female participants mentioned financial burdens (i.e., education and living costs) as a barrier to purchasing new nets and accessing health services. In order to solve these problems, some of the young female participants reported that they would seek help from their extended family.

“Many people travel on foot. If people have money, they will take a taxi boat from Port Patrick to the dispensary or they will take an airplane to the hospitals on Tanna and in Port Vila. They can borrow money from their extended families. Otherwise, they will walk or ask strong men to help.” (Female KII, Analgaut)

“Travelling by taxi boat is expensive. All my families walk to the dispensary. We have relatives living in the main village, so we can stay at their house.” (Female IDI, Port Patrick)

Because some young people on the island did not use or buy nets, many older participants emphasized the importance of information sharing for ME.

“Some young people do not buy nets. They are crazy. Information sharing is very important.” (Female IDI, a small village near Analgaut).

Written health education materials were occasionally reported as barriers to information sharing. Most participants reported preferring face-to-face communication.

“Some people do not read the notice. Picture-based instructions will help promote a better understanding. Several languages are spoken on this island. That sometimes makes things difficult. Face-to-face communication is the best way to communicate with people.” (Female KII, Analgaut)

Several informants noted the importance of education to support people who did not understand or respond to health messages. A few elderly female key informants stated that they were good performers to raise awareness.

*Leadership skills* A few elderly male key informants and interviewee explained that a decline in the population on the island due to ‘blackbirding (the recruitment of South Pacific islanders to secure cheap labour)’ in the 1800s and imported diseases undermined their *kastomary* system. An elderly male interviewee stated that chiefs tried to strengthen *kastomary* system and practices through education and awareness. Some elder key informants reported that they were eager to educate youth so that they can assume future leadership roles in the communities.

“We will prevent malaria. In the past, many people were killed or taken. Now we try to strengthen our system and develop our future leaders.” (Male KII, Analgaut)

Many informants stated that they were willing to promote community health. Some of them reported that they needed more information to be independent.

“To keep people informed is very important, then the communities can do for themselves.” (Female KII, Analgaut)

A few informants and interviewees noted that they needed training for the communities.

“I will go to school to be a nurse, because we have no female nurse on the island.” (Female IDI, Analgaut)

“Most people need money on this island, and I have five children. I work on a contract basis. I want to contribute to the communities. I need more training to become a mentor. And then I will train younger generation.” (Male KII, Analgaut)

### Participant perceptions related to social-contextual resources

Social-contextual resources consisting of ‘social services (organizational structure)’ and ‘support networks (social structure)’ promoted ME efforts.

### Social services

A local health coalition provided ME services.

#### Theme 3: An existing local health coalition

**Fig. 1:**
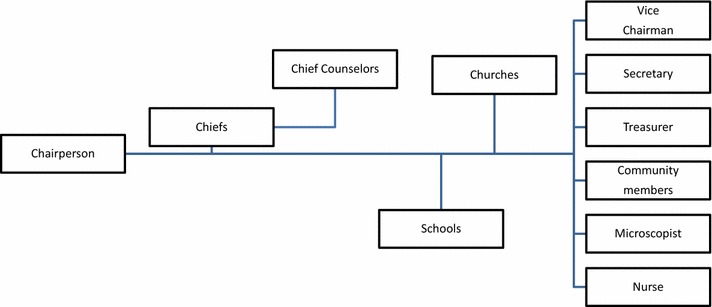
The health committee.

Within the local organizational structures of the islands (i.e., the health committee that included local authorities), a health coalition was established to provide ME services to community members (Fig. 1). These services included health promotion, awareness, education, treatment, vector control measures (such as supplementary ITN distribution, the draining of swamps and the use of larvivorous fish in ponds) and community-based surveillance. Community-based surveillance was conducted by a malaria microscopist along with the provincial malaria team. The health committee recently added a male trainee (a microscopist) to ensure availability of staff for robust surveillance. Still, several participants addressed the challenges of screening due to lack of equipment. Understaffed and undersupplied facilities were the most commonly cited barriers to providing and accessing ME services among participants. The supply ordering systems between Aneityum and Tanna Islands reportedly did not always work. Some key informants noted the importance of prevention.

“Health Facilities have no drugs, no water and no female nurse. A community radio is broken. We have many problems so prevention is very important. Prevention is better than cure.” (Female KII, Analgaut)

A retired female nurse occasionally assisted sick people. Some female participants stated that they preferred to share their health problems with female health staff. They reported that a retired female nurse was only available on the island. This finding identifies the importance of same gender health staff to establish communication channels and promote engagement.

“I talk with my husband when I have health problems. And my husband will talk to a male nurse. I do not want to talk about my health with other males.” (Female IDI, Port Patrick)

Most key informants held multiple leadership positions in their communities and some informants were closely related to one another, which reportedly strengthened a local health coalition. Faith-based activities and school-based activities promoted ME activities. Awareness campaigns were conducted by health staff, church leaders and teachers. The tidy village campaigns were mainly organized by schools to ensure cleanliness.

“We just decided to start a tidy village campaign this year. This campaign is held on Tuesdays. Households, students and parents have a clean-up day. The competition takes place every two to three months. Primary and secondary students will pick up trash and count the number. Area is divided into four parts. We manage our campaign.” (Female KII, Analgaut)

Many participants stated that chiefs promoted community health.

“When a chief blows a conch shell, people gather. He gives information to all members once in a month. Now, this village has developed. Many health staff visit this village. Chiefs work together with them.” (Female IDI, a small village near Analgaut)

Some male participants noted that local system and norms played a role in decision making process.

“My clan, the biggest clan on the island, has more than 300 members. We talk and decide everything.” (Male IDI, Analgaut)

“There are three or four tribes in my area. We share the land and live close. So we help each other to solve the health problems.” (Male IDI, Port Patrick)

“People follow their family rules and practice their religion to live a healthy lifestyle. Head of the family is not a job for a woman. Women are responsible for child-rearing, cooking and weaving. Men make the final decisions.” (Male IDI, Unmet)

### Support networks

Members of networks were sources of information and assistance for ME efforts.

#### Theme 4: Sources of information

All participants belonged to multiple support networks such as chiefdoms, lineages, kin groups, health, religion, education or sports. It was commonly stated that participants were likely to have frequent face-to-face contact with network members, and they preferred face-to-face advice.

**Fig. 2:**
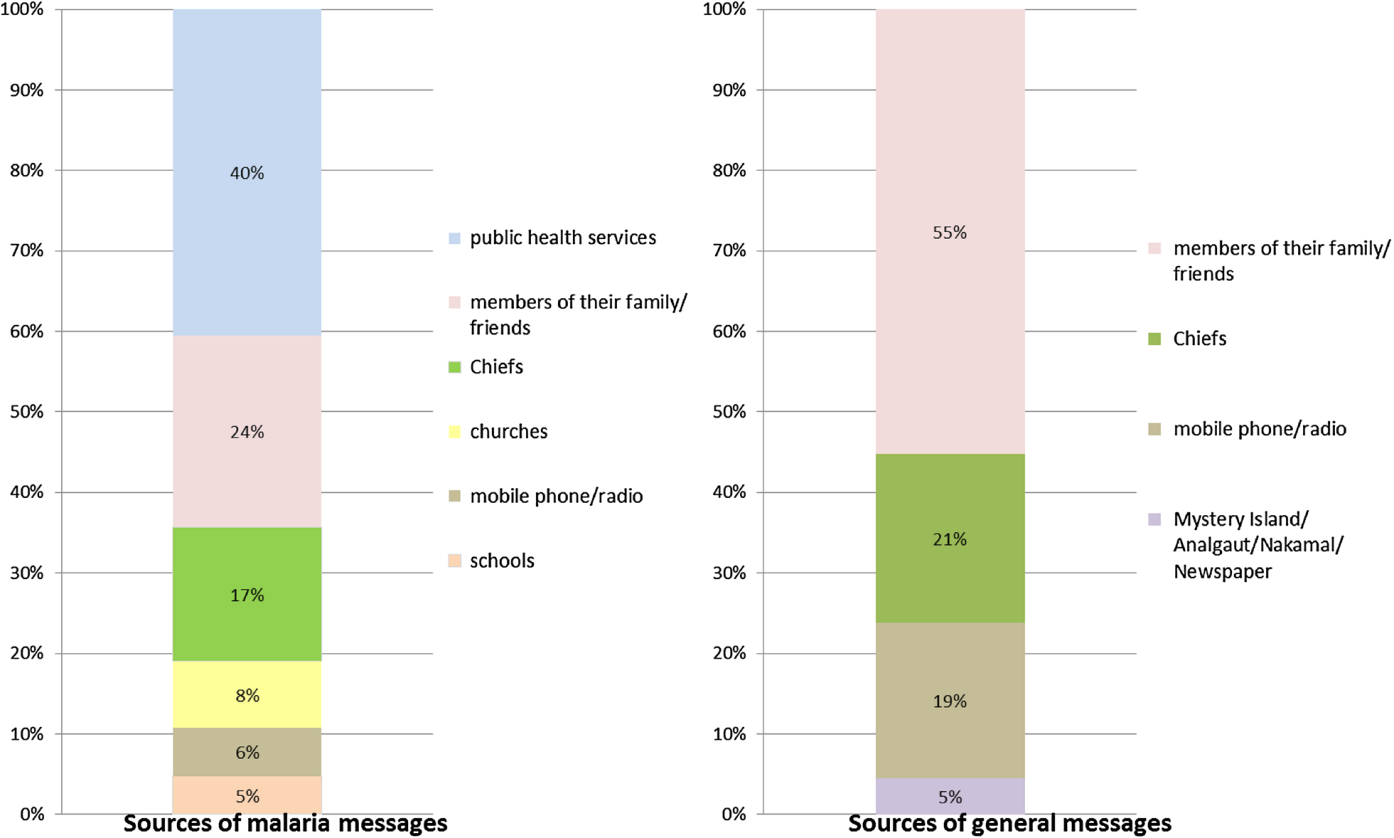
Sources of information.

Figure 2 shows that participants share information on malaria through both formal networks (e.g., public health services, chiefs, churches and schools) and informal networks (e.g., kin and friends). These networks were reportedly used for delivery, exchange and gathering. Communication channels for ME included public notice, posters, leaflets, community meeting, the *Nakamal* (a traditional meeting place or kava bar), church services, drama, events, storytelling, workshops, school curriculum, face-to-face conversations, mobile calls and text messages. Some participants owned mobile phones or personal radios. Community meetings were commonly thought to be the best way to mobilize all community members and share malaria information.

#### Theme 5: Sources of assistance

All participants reported that help was available to them from somewhere in their networks. It was commonly stated that kin in the neighborhood mutually supported one another.

“For example, a big sister will ask me to help if her daughters are unavailable. I will ask her to help me in time of need. Some people will ask their cousins or relatives. We are neighbors and friends. We support each other when someone has a health problem. This is our system.” (Female KII, Analgaut)

All IDIs reported they had three or more kin (cousins of similar age and gender, siblings, parents and grandparents) in their neighborhood (estimated to be within a five-minute walk) to turn to for help. Two female interviewees named each other as friends (kin) to count on. Supportive relationships reportedly maintained a healthy lifestyle among members.

“When I have health problems, more than ten friends in my neighbourhood support me. Friends mean kin or extended families to support one another. If I shout for help, they will come and take me to the dispensary.” (Male IDI, Port Patrick)

## Discussion

Community resources (personal and social-contextual resources) on Aneityum were identified, developed and poured into eliminating malaria by the local people in the process of community engagement. Interrelated individual and structural forms of capacity facilitated the effective mobilization and utilization of resources for their health and well-being. These multilevel empowerment responses across individual, organizational and community structural levels in community engagement continuum are explained further below in the context of HET.

### The recognition and development of personal resources

The results of this study showed that people who acknowledged the threat (severity and susceptibility) were intrinsically motivated and committed to engaging in active efforts in order to reduce importation and outbreak risk by developing individual level ME knowledge and skills, which might enhance their self-efficacy to further promote and maintain health-enhancing behaviors. People used their problem solving techniques to access, share or develop ME resources. People who experienced past malaria endemicity tended to focus their attention on sustaining engagement in ME and empowering the next generation. People who utilized the resources available for ME gained control over their behavior and their lives. These findings do not fully support previous findings, which have shown that the communities of Aneityum are motivated both by the benefits to the health of the islanders and by the fact that cruise ships call at Mystery Island and would stop doing so if malaria returned [13]. Although the sale of tourism products, such as seafood, handicrafts and docking fees were widely recognized as sources of income or sources of financial assistance for community-based activities (e.g., school fees) in this study, there is clear indication that the health of the islanders is more important than extrinsic (material) incentives.

### The provision and utilization of social services (social-contextual resources)

Communities where vector-borne diseases are endemic often lack adequate institutional systems and structures to facilitate community engagement in disease control strategies [4]. However, existing organizational and leadership structures on Aneityum facilitated community engagement in ME. A health coalition on Aneityum provided ME services, promoted collaboration between health staff and community leaders (such as chiefs, church leaders, teachers, youth leaders and group leaders) and coordinated ME activities. It generates resources for ME and facilitates the communication flow through its overlapping and interrelated networks, which increases resource mobilization and utilization on the island. In Isabel Province, Solomon Islands, a local leadership structure known as ‘the Tripod’, a cooperative alliance between village Chiefs, the provincial government and the Church of Melanesia (Anglican) along with the Mothers’ Union (an international charitable network of Anglican women), has a strong influence on communities with the spirit of togetherness in community tasks and activities for ME [3, 20]. These remote Western Pacific island communities on Aneityum Island and in the Isabel Province demonstrate that existing local organizational structures built around their norms, customs, beliefs and values foster community self-reliance and ownership of health initiatives to implement the community engagement strategies in the community-based interventions (i.e., outreach and health promotion activities and service delivery) in a locally appropriate, feasible and sustainable way.

### The recognition and utilization of support networks (social-contextual resources)

Existing networks facilitated service delivery, service utilization and resource mobilization. The more membership networks people recognized and belonged to, the more resources they could access or share. Networks of mutually supportive relationships (aligned with social capital concepts in which networks and the associated norms of reciprocity have value [21]) fostered social cohesion and solidarity in traditional communities on Aneityum through collaboration. Communities that are rich in social capital (e.g., those in the Isabel Province and on Aneityum Island) are more likely to promote health-enhancing behaviors [21, 22]. This study indicates that the existing local social capital on Aneityum continues to foster a strong sense of community among members, which contributes to solving community-identified problems to develop preventive health behaviors and create health-enhancing environments for individual and collective well-being.

### Community engagement as a ‘means’ and as an ‘end’

In analyzing changes in community engagement in ME since the 1990s, it is clear that ‘community engagement as a means’ to achieve predetermined objectives within a specified time-period facilitates ‘community engagement as an end’ where the communities sustain the capacity development process to exercise more control over their own health and environment. The means approach is usually adopted in disease control programs [4, 23, 24]. However, this study indicates that both approaches (the means and ends approaches) can coexist [24] and reinforce each other in malaria elimination program on Aneityum to sustain the changes.

### Prescription for sustaining community engagement

This study demonstrates how over time the communities have taken steps to internalize key issues and act in their own self-interest in a goal-oriented intervention (i.e., malaria elimination) for their health and well-being in the community engagement continuum. These local capacity development efforts facilitate empowerment. Community engagement produces empowerment and at the same time, it is only possible with empowerment [4]. Community engagement needs to mature into empowerment to sustain interventions [8]. Empowerment is both an outcome and a process of community engagement [9]. This process activates momentum for change. Momentum grounded in and inspired by the local context will continue to help achieve targets even after withdrawal of the external agency and promote endogenous development in a lasting, sustainable way.

To replicate the success of Aneityum on larger islands with more complex socio-cultural and environmental contexts (such as Tanna Island, Vanuatu), Atkinson et al. wrote a prescription for policy makers (e.g., government officials, health professionals and researchers) to build and maintain community engagement as a means to eliminate malaria [1]. This includes mobilizing local resources (leaders and networks), strategies to overcome gender barriers, provision of outreach to remote communities, monitoring and knowledge acquisition support through media campaigns to advice communities to eliminate malaria or address barriers to engagement [1]. Community engagement organized for the purpose of taking prescribed actions in the paternalistic approach of imposing interventions on communities through participatory methods is widely used, but actions are not always sustained due to mismatch between top-down program requirements and local contexts (custom, environment, motivation, needs and priorities) [2, 4, 6, 24]. People cannot be fully empowered by the health promoters or government officials; they can only empower themselves to leverage their own resources and make appropriate plans and decisions in the local context through a process of engagement [4, 9, 25]. The enhanced empowerment in the active community engagement continuum may facilitate a smooth transition from externally driven interventions to community-led interventions.

### Limitations

This prescription will most likely be written in flourishing traditional and religious communities that may be economically impoverished yet rich in community-based social capital through community leadership structure and densely knit support networks. Community engagement can take many forms and will work for interests in different ways in various situations. The qualitative results are limited to be generalized to the wider population of Aneityum. However, a range of perceptions and attitudes are captured to explain a health empowered community response in this study. Participant cancellation and time constraints faced by researchers have negative impacts on the validity of the results. The definitions of personal and social-contextual resources proposed by HET are used in this study. This study does not fully take into account economic issues including those associated with tourism that may also influence community engagement in ME. The impacts of globalization on social capital (negative and positive effects) should be further investigated on Aneityum. Responses and priorities may be influenced by the presence of the research team and local leaders. Some responses relating to elimination efforts may be subject to social desirability bias. There will be some degree of loss of nuances and depth because of the simultaneous and direct translation (from Bislama to English) and non-native interactions between a female researcher, local facilitators and participants, which may hinder free expression and accurate representation of views.

## Conclusions

Community engagement, which facilitates local personal and social-contextual resource development, has potential for target achievements and multilevel empowerment through community-based capacity development processes. Self-empowered communities have written and will continue to write a ‘prescription’ for sustaining high levels of engagement.

## Additional file

Additional file 1. Questions for interviews.
