# Supplementary material for: A prescription for sustaining community engagement in malaria elimination on Aneityum Island, Vanuatu: an application of Health Empowerment Theory
Source: Malar J. 2015 Jul 31;14:291. doi: 10.1186/s12936-015-0779-z (PMC4521369; doi:10.1186/s12936-015-0779-z)
Supplement: Additional file 1. — Questions for interviews. [file 12936_2015_779_MOESM1_ESM.docx]

**Additional File 1**

**A prescription for sustaining community engagement in malaria elimination on Aneityum Island, Vanuatu: an application of Health Empowerment Theory**

Authors: Noriko Watanabe^1^§, Akira Kaneko^1,2^, Sam Yamar^3^, George Taleo^3^,

Takeo Tanihata^4^, J Koji Lum^5^, Peter S Larson^6,7^, Nelma B.C. Shearer ^8^

§Corresponding author: Noriko Watanabe: [n881052@gmail.com](mailto:n881052@gmail.com)

This file includes interview questions.

**Questions**

**Focus Group Discussions (FGDs)**

1. **Personal resources**

Malaria prevention methods

Malaria risk

Malaria elimination (motivation)

1. **Social-contextual resources**

**Social services:**

ME services: access to health facilities and services

A malaria-free island and tourism

Communication channels (malaria and general issues)

**Social networks**

Support group or networks

A provider of assistance in dealing with health emergency and everyday matters

**Key Informant Interviews (KIIs) and In-depth Interviews (IDIs)**

○What is your role?

**Personal resources to engage in ME efforts**

○How do you prevent malaria?

○How do the communities prevent malaria?

○Are you afraid of malaria? Why?

○Are community members afraid of malaria? Why?

○Are you motivated to maintain elimination efforts? Why?

○Do you see any changes in community perception and practices before and after the malaria intervention?

**Social services (ME services) to promote ME efforts**

○Do you have any troubles to provide ME services in the community?

○Do you access health services and facilities?

○What obstacles do you see in accessing health facilities?

○Do you think a malaria-free island attracts tourists?

○Do you want to eliminate malaria? Why?

○Does community have sufficient information about malaria? If not, any improvements?

○What is the locally appropriate communication method?

○Where do you get the information? (malaria information and general information)

**Supportive networks**

○Please describe support group or networks.

○Whom will you ask for help (health/ general issues)?

**Asking IDIs only**

Please provide detailed information about people outside their home whom they would ask for help if it was needed in dealing with health emergency and everyday matters.

Age

Gender

Relationship

Places of residence (walking times)
